# A scoping review of nursing interventions to reduce PTSD in adolescents who have been sexually abused

**DOI:** 10.1186/s12912-024-02130-5

**Published:** 2024-07-09

**Authors:** Iyus Yosep, Suryani Suryani, Henny Suzana Mediani, Ai Mardhiyah, Taty Hernawaty

**Affiliations:** 1 Department of Mental Health, Faculty of Nursing, Universitas Padjadjaran, Bandung, Indonesia; 2 Department of Pediatric Nursing, Faculty of Nursing, Universitas Padjadjaran, Bandung, Indonesia

**Keywords:** Adolescents, Nursing intervention, PTSD, Sex Offenses

## Abstract

Incidences of sexual violence have increased over the past few years. The negative impacts of sexual violence on adolescents are social isolation, low self-esteem, and disrupting the developmental stages of adolescents, and can even cause the risk of suicide. Nurses as providers of comprehensive nursing care have a role in reviewing various aspects to reduce the impact of sexual violence on adolescents. The purpose of this study is to explore methods of nursing intervention for reducing the symptoms of post-traumatic stress disorder among adolescents who are victims of sexual violence. The design used in this study is scoping review. Article were searched from CINAHL, PubMed, and Scopus databases. The inclusion criteria for articles in this study were full text, randomized control trial or quasi-experimental research design, English language, samples is adolescents (10–19 years based on WHO) who are victims of sexual violence, and the publication period of the last 10 years (2013–2022). We found 12 articles which discussed about nursing interventions in reducing PTSD symptoms in adolescents who are victims of sexual violence. Range of the samples is 40–405 adolescents. Several articles from developed countries. There are three nursing intervention methods that can be carried out, namely improve skill interventions, relaxation interventions, and cognitive behavior therapy. Nurses act as educators, facilitators and counselors so that victims can recover from their traumatic experiences. Providing nursing interventions to adolescents who are victims of sexual violence needs to pay attention to all aspects that affect the physical and psychological condition of the victim.

## Introduction

The World Health Organization (WHO) defines sexual violence as any sexual act, attempt to obtain a sexual act, unwanted sexual comments or advances, or acts to traffic or otherwise directed against a person's sexuality using coercion, by any person regardless of their relationship to the victim, in any setting [1]. Sexual violence that is categorized as physical contact can be in the form of sexual immorality or groping the child's body, asking the child to hold or touch the perpetrator's body parts [2]. Sexual harassment and rape occur when the perpetrator has more power than the victim. Power can be in the form of a higher job position, economic power, the power of one sex over another, a greater number of personnel, and so on [3].

Data recorded according to the World Health Organization estimates that in 2017, there are around 1 billion children aged between 12–17 years who have experienced physical, emotional and sexual violence [4]. According to a report by the United Nations Children's Fund (UNICEF) in 28 countries, there were 2.5 million young women who reported having received acts of sexual violence, either through physical contact or not before the age of 15, in India there were 7,112 cases, Zimbabwe in 2011 reached 3,172 cases, England in 2012 reached 18,915 sexual crimes against children [5]. Likewise sexual violence against children, there were 250 cases of sexual violence against children where these cases have increased compared to previous years [6].

Sexual harassment can have a profound traumatic effect on the victim. Victims of sexual harassment can experience stress due to the traumatic experience that occurred at the time of the incident [7]. Victims of sexual harassment and rape often experience Post Traumatic Stress Disorder (PTSD), characterized by anxiety, emotional instability, autonomic lability, and painful flashbacks. This disorder results from traumatic experiences that exceed typical resilience levels [8].

PTSD is a syndrome of anxiety, autonomic lability, emotional invulnerability, and flashbacks of very painful experiences after experiencing physical or psychological stress beyond the limits of ordinary people's endurance [9]. PTSD is very important to know, apart from the many incidents or disasters that have befallen us, PTSD can also affect anyone who has experienced a traumatic event regardless of age and gender [10]. Victims of PTSD show behavioral disturbances in social life in the form of panic attacks, withdrawing and avoiding groups, depression, playing or trying to get rid of thoughts and feelings, feeling left out and alone, feeling insecure and betrayed, easily angry and assuming distractions in everyday life [11, 12].

Previous systematic review have shown that nursing interventions given to victims of sexual violence can effectively reduce anxiety symptoms [13]. This study is less effective for some people because it already has a negative impact, namely PTSD. So, the study recommended to make a review of nursing interventions to reduce PTSD symptoms in adolescents who experienced sexual violence. Another study showed that nursing interventions can effectively reduce symptoms of low self-esteem in victims of sexual violence [14, 15]. Several samples had problems in the intervention process because it had a post-traumatic effect due to sexual violence. This study also recommends conducting a review of interventions to reduce the effects of trauma on adolescents as victims of sexual violence. So, this study is the first scoping review that describes nursing interventions to reduce PTSD symptoms in adolescents who are victims of sexual violence.

Nurses act as counselors and advocates in dealing with PTSD problems in victims of sexual violence. However, previous study have shown that nurses are not optimal in providing comprehensive nursing care to victims of sexual violence [16]. Nurses are still focused on handling medical problems experienced by victims so they are lacking in dealing with long-term effects, namely PTSD. The purpose of this study is to describe nursing intervention methods to reduce PTSD symptoms in adolescent victims of sexual violence.

## Material and methods

### Design

This study used design scoping review. Scoping review have the aim for exploring topics that are developing in the present [17]. This study was chosen by the authors because it has a wide conceptual range to comprehensively discuss the research objectives [18]. The stages of this study consisted of determining research objectives, preparing research questions, determining inclusion and exclusion criteria, selecting articles, and preparing reports on the results of the analysis in scoping review [19]. This study used PRISMA Extension for Scoping Reviews (PRISMA-ScR) as a strategy to identify and select articles that discuss nursing interventions to reduce PTSD symptoms in adolescents who are victims of sexual violence [20].

### Search methods

Articles used in this study were from three databases: CINAHL, Pubmed, and Scopus. The keywords used are: “nursing care OR nursing intervention” AND “sexual offenses OR sexual violence OR sexual abuse OR sexual harassment OR sexual assault” AND “Post-Traumatic Syndrome Disorder OR PTSD” AND “adolescents”. The question in this study is what the methods of nursing intervention can be used to reduce PTSD symptoms in adolescents who are victims of sexual violence?

### Inclusion and exclusion criteria

Studi ini menggunakan eligibility criteria yaitu PCC’s (Population, Concept, Context) framework:

Population: adolescents (10–19 years based on WHO) [21].

Concept: Nursing Interventions.

Context: Post-traumatic stress disorder among adolescents who victims of sexual violence.

**Fig. 1:**
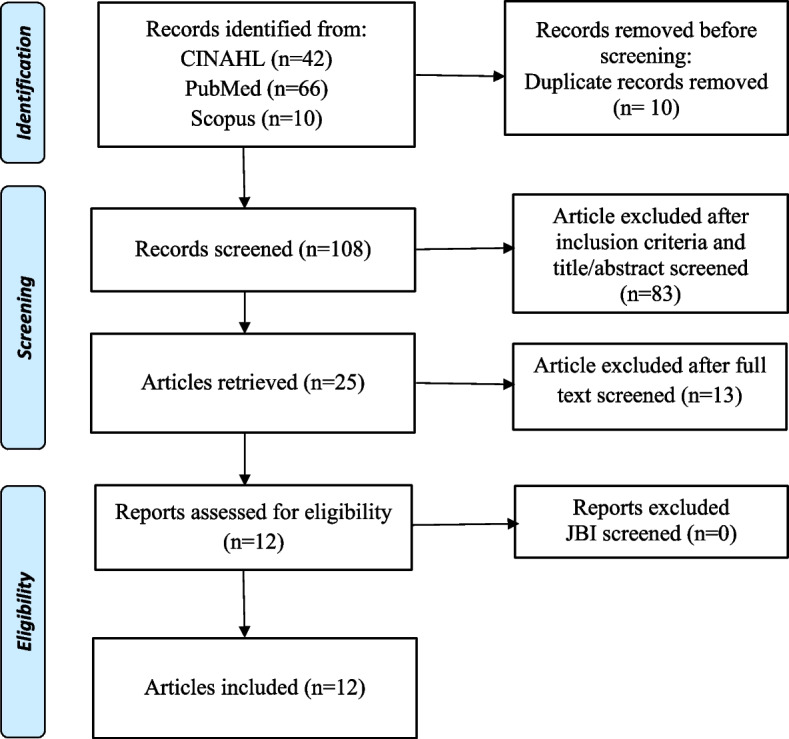
PRISMA flow diagram

The search strategy used in this study is PRISMA Extension for Scoping Review (PRISM -ScR) to identify types of nursing interventions to reduce PTSD symptoms in adolescents who are victims of sexual violence (Fig. 1). The inclusion criteria and exclusion criteria were used for the selection of articles in this study. The inclusion criteria used in this study were that the sample was adolescents, the intervention was carried out by nurses, using English, full text, using a randomized control trial design or quasi experiment, and the time setting was the last 10 years (2013–2022). While the exclusion criteria in this study were interventions not to reduce the impact of PTSD and the sample was not adolescents.

### Data extraction

The authors extracted data using manual tables created by the authors. The extraction table aims to analyze study results based on important aspects of the reviewed articles easily. So that the writer can compare and classify every aspect of the results of the articles that have been reviewed. The aspects that the authors recap on the extraction table are the author, year, country, research design, sample, intervention, and results of the study.

### Quality appraisal

The assessment of article quality used the Joanna Brigs Institute (JBI) instrument to determine the quality of the articles analyzed in this study. The JBI assessment method uses statements assessed by the authors according to the guidelines from The Joanna Brigs Institute (JBI). Statements for randomized control trial designs consist of 13 JBI instrument statements and 11 statements for articles with quasi-experimental designs. The score for each statement is yes, no, unclear, and not applicable. The assessment of each score, namely the "yes" score, is given a value of 1, while the scores for "no" and "unclear" are given a value of 0. The standard JBI assessment score used for the articles analyzed in this study is above 75% (Table 1).

**Table 1:** JBI critical appraisal tool

Author, Published Year	JBI Critical Appraisal Tool	Study Design
Raabe et al. 2015 [22]	84,6% (11/13)	RCT
Pyne et al. 2019 [23]	92,3% (12/13)	RCT
Daneshvar et al. 2022 [24]	76,9% (10/13)	RCT
Rajan et al. 2022 [25]	76,9% (10/13)	RCT
Belleville et al. 2018 [26]	92,3% (12/13)	RCT
Gilmore et al. 2021 [27]	84,6% (11/13)	RCT
Jimenez Peregrina et al. 2020 [28]	84,6% (11/13)	RCT
Andersen et al. 2021 [29]	92,3% (12/13)	RCT
Orang et al. 2018 [30]	76,9% (10/13)	RCT
Pearson et al. 2019 [31]	76,9% (10/13)	RCT
O’Cleirigh et al. 2019 [32]	84,6% (11/13)	RCT
Murray et al. 2018 [33]	92,3% (12/13)	RCT

### Data analysis

This study used a descriptive approach to data analysis. The authors selected articles based on inclusion criteria and exclusion criteria to be presented in the study report. The authors read the article as a whole and then makes a summary of the contents of each article. The data obtained from the results of article reviews were then analyzed by the authors based on similar characteristics. Then the authors made a description of nursing interventions to reduce PTSD symptoms in adolescents who are victims of sexual violence. After that, the authors classified nursing intervention methods based on similar methods.

## Results

The authors found 118 articles based on an initial assessment search from three databases, namely CINAHL, PubMed, and Scopus. After that, the authors made a selection based on duplication of articles using the Mendeley application, the authors obtained 108 articles. Then the authors eliminated based on the inclusion criteria and read the title and abstract, the results of the elimination showed that there were 25 articles. Then the authors read the full-text articles and discusses the results of the article review to determine which articles are relevant to the research objectives. The authors obtained 12 articles that are in accordance with the research objectives. The authors evaluated the articles using the JBI Critical Appraisal Tool to ensure that the articles used in this study are of high quality. The article used in this study has a standard value for the JBI assessment, which is above 75% (Table 1).

There were 12 articles that discussed nursing interventions to reduce PTSD symptoms in adolescents who were victims of sexual violence. The authors has read and analyzed the 12 articles obtained. We classified it into three intervention methods, namely Improve Skill Interventions, relaxation interventions, and cognitive behavior therapy. The results of the analysis of the article are presented in manual form from all authors presented as follow (Table 2):

**Table 2:** Extraction data

**The explanation of each nursing intervention method**			
**Authors and Year**	**Purpose**	**Method**	**Result**
**Improve Skill Interventions**			
Raabe et al. 2015 [22]	Reduce negative effect of traumatic experience	STAIR is an intervention that is carried out online. Participants carry out activities for 12 weeks (30 min/session by telephone). In the first session, participants were given training on problem solving. Then the participants were given time to consult with a therapist who is a mental nurse to control the trauma experienced by victims of sexual violence	there was a decrease in PTSD symptoms in victims of sexual violence
Pyne et al. 2019 [23]	Improve resilience and reduce PTSD on victims of sexual violence	Heart Rate Variability and Cognitive Bias Feedback Interventions are cognitive capacity building activities to overcome the effects of trauma. Participants take part in activities for 16 weeks (1 h/session). Participants were given psychoeducation about trauma management which can be done independently for 40 min. Then participants will conduct training sessions to improve empathy, problem solving, and emotional management	Significant decrease in PTSD symptoms in victims of sexual violence
Rajan et al. 2022 [25]	reduction of symptoms of PTSD in individuals with PTSD	Modified Lifespan Integration is an intervention carried out over 8 sessions. Each session is given time for 90–140 min. The intervention was only carried out by female therapists because most perpetrators of sexual violence were men, which traumatized victims. The sessions are audio-recorded and stored to allow monitoring of therapist adherence to treatment protocols. Participants will be trained to express their feelings and then be directed to make several solutions to the problem of trauma due to sexual violence	effective results in reducing PTSD symptoms
Orang et al. 2018 [30]	Effect intervention on psychological wellbeing and PTSD	Narrative exposure therapy is an intervention in the form of consultation to seek help from resources such as family of origin, police or lawyers, improvement of coping skills and safety planning, and human rights education. Participants intervened for 10 sessions with a duration of 120–150 min/session. The first session began with a discussion between participants and nurses about the sexual violence they experienced, to make sure the patient was ready to face traumatic events in the past. Then participants took part in a psychoeducational session about PTSD symptoms and an explanation of goals and interventions. Then the participants attended counseling with psychiatric nurses and psychologists to resolve the trauma they experienced	This intervention has been shown to reduce PTSD symptoms in victims of sexual violence
Gilmore et al. 2021 [27]	Reduce symptoms of PTSD	Video-Based Intervention features a female narrator who provides information to prevent emotional problems in victims of sexual violence. The videos provide instructions for implementing self-help exercises for dealing with trauma, methods for recognizing and stopping inappropriate coping, and strategies for engaging in activities that reduce PTSD. The intervention was carried out for 12 weeks for 8 sessions	This intervention can effectively reduce PTSD symptoms in victims of sexual violence
**Relaxation interventions**			
Jimenez Peregrina et al. 2020 [28]	Improve resilience and reduce symptoms of PTSD	Eye movement desensitization and reprocessing (EMDR) therapy is a comprehensive patient-centered psychotherapy approach over 8 sessions(Jimenez Peregrina et al., 2020). EMDR therapy is guided by nurses to control the emotions felt when remembering traumatic experiences. Participants were asked by the nurse to remember the traumatic experiences experienced by the participants. Then participants perform eye movements to address the memory you are targeting. During this session, participants were asked to focus on negative thoughts, memories, or images they were experiencing	The results of this intervention show a decrease in PTSD symptoms in victims of sexual violence
Andersen et al. 2021 [29]	Effect on psychological and somatic symptoms, and reduce PTSD	The Mindfulness-Based Stress Reduction (MBSR) intervention is a therapy that is carried out for 8 weeks (2 h/week). Activities undertaken include experiential exercises on non-judgmental mindfulness of breathing, body scanning, and yoga. Nurses play a role in training participants to relax and guide the course of mindfulness therapy	This intervention has been proven to reduce symptoms of trauma experienced by participants who experienced sexual violence
**Cognitive behavior therapy**			
Belleville et al. 2018 [26]	Reduction of symptoms of PTSD	Imagery Rehearsal Therapy and Cognitive Behavioral Therapy is carried out for 15 sessions/15 weeks. The activities carried out in this intervention are psychoeducation, counseling, and skills training such as empathy, problem solving, self-management, and emotional management. The nurse acts as an educator to increase participants' understanding of trauma, its causes and effects. Nurses also act as trainers to improve participants' skills in overcoming PTSD	This study shows that the intervention is proven to be effective in reducing PTSD symptoms
Daneshvar et al. 2022 [24]	Improve meaning-in-life and reduce PTSD	Group-based Compassion-focused Therapy is an intervention that is carried out offline in 8 sessions (1 h/session). Activities in this intervention started with psychoeducation about trauma and the effects of trauma. Then participants take part in activities to train empathy, train sympathy, practice forgiveness, practice acceptance of unpleasant events, train to develop feelings of worth and transcendence: Helping individuals to develop feelings of self-esteem so they can deal with conditions effectively and efficiently, train commitment to self themselves, and practice and review previously taught skills. Nurses act as facilitators and educators in the training process carried out	This study shows that there is an improvement in mental health and a decrease in PTSD symptoms
Pearson et al. 2019 [31]	Effect intervention on PTSD symptoms, high-risk sexual behavior, and substance use	Cognitive Processing Therapy was carried out in 13 sessions for 16 weeks. Activities carried out are identifying the worst trauma and reviewing symptoms of trauma, overcoming "unhelpful" mindsets, "balancing" thoughts and feelings, increasing trust, power and control, respect, and caring. The nurse acts as a facilitator to guide the intervention process	This intervention is proven to reduce the symptoms of trauma experienced by participants
O’Cleirigh et al. 2019 [32]	Effect on PTSD symptoms	Cognitive Behavioral Therapy for Trauma and Self Care focuses on counseling to reduce the impact of sexual violence experienced by participants. The intervention was carried out for 8 sessions for 16 weeks. Participants were given psychoeducation about the effects of trauma, normalizing symptoms of post-traumatic stress, and reviewing the adaptive coping strategies used. Then participants were given cognitive therapy strategies, behavioral techniques, and counselling. Then participants were given lessons on identifying cognitive distortions (eg self-blame, guilt), and strategies for challenging and changing unrealistic beliefs	Effectively reduce symptoms of PTSD on victims of sexual violence
Murray et al. 2018 [33]	Effect on PTSD and anxiety	Cognitive processing therapy (CPT) is psychotherapy to reduce mental health problems in victims of sexual violence. Treatment occurred over 12 sessions (with a duration of 2 h/session). The activities carried out were psychoeducation about PTSD and how to deal with trauma due to sexual violence, then participants were given training in empathy and problem solving skills to discuss how to deal with the effects of PTSD. This study shows that CPT can reduce PTSD symptoms	Significantly reduce symptoms PTSD and anxiety

Based on the results of a study on research sites, 3 articles came from the USA, 1 article came from Colombia, 1 article came from China, 1 article came from New York, 1 article came from Germany, 1 article came from Sweden, 1 article came from Mexico, 1 article is from Norway, and 1 article is from France. Based on country characteristics, all articles come from developed countries. Based on the characteristics of the sample in the article, the range of the samples is 40–405 adolescents. The sample is adolescents with an age range of 11–25 years who are in the developmental stages of adolescence and early adulthood.

The results of this scoping review show that nursing interventions can reduce PTSD symptoms in adolescents who are victims of sexual violence. There are three nursing intervention methods that can be used, namely Improve Skill Interventions, relaxation interventions, and cognitive behavior therapy. Activities carried out from nursing interventions are generally psychoeducation, increasing skills such as empathy, problem solving, and sympathy, as well as relaxation to make peace with unpleasant events.

Activities in Improve Skill Interventions include STAIR [22], conducted online over 12 weeks, Heart Rate Variability and Cognitive Bias Feedback over 16 weeks [23], and Modified Lifespan Integration over 8 sessions session [25]. These methods involve training in skills such as empathy and problem-solving, as well as consultations with therapists, and have been shown to effectively reduce PTSD symptoms. Narrative Exposure Therapy, consisting of 10 sessions [30], and Video-Based Intervention over 12 weeks also demonstrated a reduction in PTSD symptoms through counseling and self-help education [27].

Relaxation interventions such as Eye Movement Desensitization and Reprocessing (EMDR) over 8 sessions [28] and Mindfulness-Based Stress Reduction (MBSR) over 8 weeks [29], involve relaxation and mindfulness techniques that effectively reduce trauma symptoms. Cognitive behavior therapy including Imagery Rehearsal Therapy and Cognitive Behavioral Therapy over 15 sessions [26], Group-based Compassion-focused Therapy over 8 sessions [24], Cognitive Processing Therapy over 13 sessions [31], and Cognitive Behavioral Therapy for Trauma and Self Care over 8 sessions [32], all involve psychoeducation, counseling, and skills training [33], and have proven effective in reducing PTSD symptoms in victims of sexual violence.

## Discussion

This scoping review showed that 12 articles discussed about nursing interventions to reduce symptoms of PTSD in sexual violence on adolescents. Each intervention has been proven to reduce PTSD symptoms in victims of sexual violence using several methods. There are three nursing intervention methods, namely improve skill interventions, relaxation interventions, and cognitive behavior therapy. The nursing interventions provided pay attention to the characteristics of the participants so that the results of the intervention are achieved optimally.

Nursing interventions are crucial in reducing PTSD symptoms in sexual assault victims due to their holistic and personalized approach to care. These interventions are important because they address both the immediate psychological trauma and the long-term mental health needs of victims, which are often overlooked [34]. Nurses have an important role in these interventions, acting as facilitators, educators, and supporters throughout the recovery process. Nurses can provide psychoeducation, emotion regulation training, and skills development, tailored to the individual needs of each victim [35]. The integration of nursing interventions offers a more comprehensive and supportive framework for survivors of sexual violence, ensuring that they receive ongoing, compassionate, and specialized care [36].

Nurses have a role as facilitators, advocates, educators, and counselors in conducting interventions for victims of sexual violence who experience PTSD. Nurses become facilitators in guiding the interventions given to participants. Then the nurse becomes an advocate to help handle cases of sexual violence experienced by victims by collaborating with related parties. The role of the nurse as an educator is given to victims to find out about independent interventions and prevent worse psychological impacts on victims of sexual violence [37, 38]. Nurses collaborate with psychologists to provide counseling to victims of sexual violence, nurses help patients recover from their trauma by discussing the best solutions with participants. Providing nursing interventions is carried out in a comprehensive manner by looking at aspects that affect the physical and psychological condition of the victim [36, 39, 40]. The focus of the nursing intervention given is that victims feel safe and comfortable to reduce the PTSD symptoms they experience. So nurses need to do research on the victim's condition before giving nursing interventions. This is in line with previous studies which show that giving interventions to victims of sexual violence must pay attention to the characteristics of the victim to increase the victim's self-confidence [28, 41]. Other study showed that there is a relationship between cultural approaches in improving the mental health of victims who experience sexual violence [42, 43].

Skill Interventions are interventions that focus on increasing the victim's ability to reduce PTSD symptoms in victims of sexual violence. The psychological skills needed by victims of sexual violence are resilience, adaptive coping, problem solving, and decision making [44]. This intervention involves the family and victims so that therapy can be carried out at home. This capability aims to increase the awareness of victims and also their families to overcome the trauma experienced by victims of sexual violence [45]. Skills improvement interventions affect the ability of sexual victims to find solutions to reduce the PTSD symptoms they experience. Victims are assisted by nurses to find solutions in dealing with trauma.

Skill improvement interventions carried out by nurses in groups [46]. Participants also carry out individual counseling to find out the problems faced by victims of sexual violence. This study shows that this intervention is effective in reducing PTSD symptoms in victims of sexual violence. This activity requires full supervision by the nurse to ensure the success of the intervention provided. A previous study conducted by Tasca et al., (2016) showed that there was a high effect of resilience and problem solving on victims of sexual violence with a decrease in PTSD symptoms in victims of sexual violence [47].

Relaxation interventions are interventions that focus on increasing relaxation in reducing PTSD symptoms in victims of sexual violence. This activity is carried out by calming the patient so that they focus on reducing PTSD symptoms in victims of sexual violence. Previous study on relaxation techniques, namely Eye Movement Desensitization and Reprocessing (EMDR) are therapies that use voluntary back and forth eye movement to reduce anxiety related to thoughts that bother PTSD patients [48, 49]. This therapy focuses on images of the trauma as well as negative thoughts and affective responses elicited by the trauma. The goal of this therapy is for a person to think and be more positive about the trauma they are experiencing [35, 50]. EMDR uses bilateral stimulation in the form of saccadic eye movements or other alternating eye stimulation, carried out while exposed (focusing on disturbing memories, emotions and cognitions). This therapy has proven effective in reducing PTSD symptoms in victims of sexual violence. Another study showed that relaxation can reduce anxiety in victims so that victims focus on discussing with psychiatric nurses in reducing the effects of the trauma they experience [51, 52]. However, other study also showed that relaxation techniques only reduce PTSD symptoms temporarily, so a follow-up period of 1 year is needed to optimally reduce PTSD symptoms [27]. This shows that relaxation interventions require longer supervision and assistance by mental nurses.

The techniques used in cognitive behavior therapy carried out by nurses include cognitive restructuring, namely removing or changing one's irrational thoughts to become rational and eliminating trauma, especially anxiety and fear that has not yet happened. The stages carried out in cognitive therapy are identifying problems and looking for the most basic causes of these negative feelings, then simplifying these problems into smaller ones so they can be more easily dealt with sexual harassment experience [13]. Cognitive therapy has an effect on the mental recovery of victims of sexual harassment, this is because cognitive therapy helps victims to recover by changing their mindset or point of view so that this mindset will influence the next action or behavior of the victim [33]. This positive behavior and mindset will become a habit that is better than before, so that the commitment and support from the family or environment around the victim is very influential in changing the victim’s self [53].

CBT intervention facilitates the emotional process by helping the patient to react with some fear to the memory or recollection of the event experienced. Previous studies have shown that cognitive behavioral therapy is effective in reducing PTSD symptoms in victims of sexual violence. A previous study conducted by Botsford et al., (2019) compared prolonged exposure with cognitive processing therapy (combining exposure in the form of writing and reading about trauma and cognitive therapy) to minimize attention [54]. This study shows that both therapies are effective in reducing PTSD symptoms in victims of sexual violence.

Nursing interventions carried out for victims of sexual violence must pay attention to the physical and psychological conditions of the victim. This is due to the fact that the victim’s condition affects the intervention process carried out by the nurse. This implementation also requires collaboration with parents and psychologists to optimize the goals of nursing interventions in reducing PTSD symptoms [36]. Of the three methods obtained from the results of this study, cognitive behavior interventions are the most effective interventions for reducing PTSD symptoms in victims of sexual violence. This is because cognitive behavior is more effective and efficient and the intervention process is carried out intensively with mental nurses [7]. So that the intervention process runs effectively and there is a significant reduction in PTSD symptoms in victims of sexual violence [55].

### Limitations

The limitation in this study is the limited article study design with quasi experiments and randomized control trials. Although it is intended that the articles reviewed in this study have high and good quality so that they can be an illustration in carrying out nursing interventions. Another limitation in this study is that the articles reviewed were limited to the last 10 years so that the number of nursing interventions discussed totaled 11 articles. This causes the results of the study do not broadly explain nursing interventions that can be done to reduce PTSD symptoms in victims of sexual violence.

## Conclusions

This scoping review shows that nursing intervention is one of the interventions in reducing PTSD symptoms in victims of sexual violence. The implementation of the intervention focuses on the traumatic effects experienced by the victim by taking into account the victim's condition holistically. The authors found three nursing intervention methods, namely Improve Skill Interventions, relaxation interventions, and cognitive behavior therapy. Efforts to reduce PTSD symptoms are carried out by means of psychoeducation, improving skills such as empathy, problem solving, and sympathy. Nurses as counselors, advocates and facilitators together with other health workers conduct interventions to improve the mental health of victims of sexual violence.

The implication of this study is that there is a foundation for the government in providing policies to reduce the negative impacts of sexual violence. This study can also be a recommendation for nurses who will provide nursing care to victims of sexual violence. Recommendations for further research are that a systematic review and meta-analysis is needed to measure the effectiveness of nursing interventions in reducing PTSD symptoms in adolescents who experience sexual violence.

## Data Availability

The data that support the findings of this study are available from the corresponding author, [IY], upon reasonable request.
